# From little things, big things grow: a local approach to system-wide maternity services reform in the absence of definitive evidence

**DOI:** 10.1186/1743-8462-4-18

**Published:** 2007-09-30

**Authors:** Anne-marie Boxall, Kathy Flitcroft

**Affiliations:** 1 Australian Health Policy Institute, The University of Sydney, Sydney, Australia; 2 School of Public Health, The University of Sydney, Sydney, Australia

## Abstract

**Background:**

For nearly two decades calls have been made to expand the role of midwives within maternity services in Australia. Although some progress has been made, it has been slow and, at system-wide level, limited. There are many barriers that prevent the expansion of midwifery-led services in Australia including funding arrangements for midwifery care, a lack of political will and resistance from powerful medical interest groups. The ongoing debate that exists about the evidence for the safety of midwifery-led care, particularly for the intrapartum phase, is likely to be an important reason why policy-makers are reluctant to implement system-wide reforms of maternity services.

**Discussion:**

Those opposed to the expansion of midwifery-led care argue that these services are only appropriate for low-risk women. They claim the evidence in support of midwifery-led care has too many holes in it to guarantee that services are safe for higher risk women. Midwifery advocates, however, argue there is no evidence to support the claim that midwifery-led services lead to poorer outcomes in any risk group. Despite this, funding for midwifery-led care outside hospitals remains limited. This article contends that calls for the system-wide expansion of midwifery-led care (such as through funding independently practising midwives) based on the available evidence are unlikely to succeed. There are too many methodological challenges in this area to ever "prove" that midwifery-led services are safe – except for the lowest risk women – and when there is doubt, policy-makers are likely to err on the side of caution.

**Summary:**

In order to expand access to midwifery care, advocates should abandon the idea of system-wide reform for now. Instead, they should concentrate on implementing small-scale, locally based changes because it is at this grass roots level that health professionals can work together to resolve the major sticking points – accurately assessing risk, identifying when it changes and responding appropriately. While a lack of political will is a major obstacle to reform it *is *amenable to change. We argue that system-wide reform is most likely to occur when policy-makers can reference examples of successful locally-based midwifery-led programs across Australia.

## Background

The term midwifery-led care can be confusing as midwifery is practised in various settings – hospitals, birth centres and community clinics – and is organised in different ways – including standard midwifery, team midwifery and caseload (or one-to-one) midwifery. In this paper, midwifery models of care are considered to be those that involve midwives as primary carers for women in at least one of the perinatal stages.

There have been many state and federal reports and inquiries into maternity services in Australia [1-6]. One of the first and most influential was the report of the *Ministerial Taskforce on Obstetric Services in NSW *[1](known as the Shearman Report). It recommended granting hospital visiting rights to suitably qualified, independently practising midwives as a means of expanding access to midwifery-centred maternity care in NSW [7]. It also suggested expanding access to birth centres in order to "fulfil women's desire for a less medicalised approach to childbirth without sacrificing the benefits which medical advances have made possible"[1].

Since then, the National Health and Medical Research Council (NHMRC) has also reviewed midwifery services. Their report, entitled *Review of Services Offered by Midwives *[2] examined evidence from nine randomised trials, including three from Australia, and noted the findings of recent reviews of birth services in NSW, Victoria and Western Australia. All favoured the introduction of midwifery-led models, which had been found to lead to lower rates of medical intervention during birth and greater reported satisfaction with care, both of which are highly valued by many women. The NHMRC report concluded that there was sufficient evidence from both international and national literature and experience to justify support for the introduction of midwifery models of care. To make this a realistic option in Australia, however, it pointed out that midwives would need to be given legal responsibility for ordering tests and initiating drugs [2].

Despite the evidence in favour of midwifery care and high-level backing from various bodies, access to midwifery care remains limited in Australia. It is especially problematic in rural areas because many small maternity units, which were staffed by midwives and general practitioners, have been closed in the past two decades. In NSW alone, 32 out of 67 rural maternity units have closed since 1995 [8]. Women are often required to travel long distances to access any form of maternity care, let alone midwifery care [9]. The perceived failure of federal and state governments to address maternity services issues has spawned a new political party in Queensland, which aims to field candidates in both houses of the federal parliament. According to party leader Justine Caines, the What Women Want Party believes "the states share the same issues – soaring caesarean rates, the closure of rural maternity units and absolutely no choice for women" [10].

The What Women Want Party is not the only group interested in expanding access to midwifery care in Australia. The Maternity Coalition Inc. is a national umbrella organisation for midwives and mothers. They argue that community midwifery is commonly practised in other countries, including the United Kingdom, New Zealand and the Netherlands, and services are easily accessed there through public health systems. In 2002, they published a *National Maternity Action Plan *(NMAP) that called on federal and state/territory governments to radically reform the way maternity services are both funded and delivered in Australia. They argued in particular that governments "work as a matter of priority towards ensuring women have universal access to primary midwifery care"[11].

In this paper, we argue that such calls for wide-scale reform are unlikely to succeed because there are too many barriers to reform in Australia. Amongst them are the prohibitive costs of personal indemnity insurance for midwives working outside the public hospital system. A further disincentive is the exclusion of independent midwifery services from the Medical Benefits Schedule, so that women using these services must pay the full cost with no government funding contribution. There has also been a history of hospital-based childbirth in Australia since the Second World War, which the medical profession dominates (both numerically and hierarchically). Its position has been reinforced by recent advances in technology and the greater reliance on hospital-based tests and interventions in maternity care. The medical profession also has a powerful and well-established representative group (RANZCOG – the Royal Australian and New Zealand College of Obstetricians and Gynaecologists) that capably protects their interests. Aside from the difficulties within maternity services itself, major health policy reforms in Australia are always complicated because responsibility and financing is shared between federal, state and territory governments.

Some commentators argue that lack of political will is the main reason there has not been a system-wide response to improve access to midwifery-led care in Australia. In 2005–06, the Maternity Coalition Inc. produced a Campaign Kit designed to influence the federal government on funding for maternity services. It argued specifically for the introduction of tied grants for community-based midwifery care as part of the Australian Health Care Agreements and a Basic Birth Care Provider Payment made available to both doctors and midwives through the Medicare Benefits Schedule. In their briefing to the federal government they argued:

*Community groups and caregivers are unanimous on the need for reform of Australia's maternity care services. Consumers and midwives have developed a tested and viable vision of where we need to go. This vision has received strong support from some areas of the medical community who recognize the evidence based benefits to women from having access to continuity of care by midwives during pregnancy and birth, with referral to medical care only if needed. The missing element has so far been the political leadership to bring about reform. We urge you to take this role *[12].

We do not disagree that political will is an important factor in the reform process but argue that uncertainty surrounding the evidence on the safety of midwifery-led care, especially during the intrapartum phase, is an important underlying reason for policy-makers' reluctance to implement major reforms. Evidence on the safety, popularity and feasibility of midwifery models of care for low-risk women is solid [13-15]and largely uncontested by medical professionals. It is the evidence on the safety of midwifery-led care for higher risk women, however, that brings the debate on expanding access to midwifery care to a standstill.

Groups such as RANZCOG accept that "some women who have been carefully assessed as being at lower risk of pregnancy complications will choose to labour in relatively low-technology primary care units". However, they are quick to point out that just because childbirth in Australia "has never been safer" it "does not mean it is without risk of serious complications". They say that because complications often occur "with frightening rapidity" and there "is at times no margin for unnecessary delays", birth centres should be co-located with 24-hour obstetric facilities wherever possible [16].

On the other side of the debate, groups like the *Maternity Coalition Inc*. argue that "it is time to break the current doctor-led monopoly on childbirth by giving midwives their proper place in maternity services". They state that "medical evidence supports the good outcomes achieved in midwifery led units and birth centres" and that "general practitioners and specialist obstetricians should not have the sole right to provide basic maternity care" because it denies women choice and "their basic human rights". They claim that women object to the domination of maternity services by medical practitioners because they are aware that this exposes them to higher risk of "unwanted medical intervention"[17].

At the heart of the debate about expanding access to midwifery care is the issue of risk and the available evidence cannot help resolve this problem. While many advocates protest that the evidence base underlying obstetric-led care is rarely questioned, this is true for many areas of conventional practice. The burden of 'proof' mostly rests with those seeking to reform the delivery of health services, particularly when they threaten to upset the established practice of medicine. As it stands, the evidence base for midwifery-led care is strong. No studies have shown any increased risk of perinatal mortality or maternal complications associated with midwifery-led care, and none have shown that midwifery-led care had a detrimental impact on other neonatal outcomes, such as Apgar scores at 1 and 5 minutes post birth or birth weight. Some studies, in fact, have found that babies born in midwifery-led services were less likely to need resuscitation [18], and that fewer were admitted to NICU following birth [15,18-20]. Despite this, those who oppose proposals for expanding access to midwifery care still refer to evidence to support their case. This paper looks at the evidence base in one of the most controversial areas of midwifery care – birth centres – and explains why it does not help put an end to the argument about the safety of midwifery care.

## Discussion

### How useful is the evidence? A case study on the safety of birth centres

Despite the evidence in favour of expanding access to midwifery-led care, critics continue to argue that it is not safe. Their claims tend to focus on the evidence concerning maternal and neonatal safety in birth centres. The highest level of evidence available is a 2005 Cochrane Review of birth centres. In this review of six randomised controlled trials (RCTs) involving 8,677 women, outcomes in home-like care were compared to those in conventional labour wards. They found that women delivering in a home-like setting were significantly more likely to require no intrapartum analgesia/anaesthesia, have a spontaneous vaginal birth, choose the same setting again, be satisfied with intrapartum care, initiate and continue breastfeeding, and have perineal/vaginal tears, but not episiotomies. Importantly, there was also a non-significant trend towards higher perinatal mortality in home-like settings [21], which is enough to sow the seed of doubt in some critics' minds.

This systematic review is particularly useful in highlighting the weaknesses in the evidence base for midwifery care. One is the definition of what constitutes midwifery-led care, as it tends to vary from study to study. While all the studies included in the Cochrane review offered intrapartum care in a home-like setting, some birth centres provided a high level of continuity of care over the antenatal, intrapartum and postnatal period [22-24], but others did not [25]. Some birth centre models had the routine involvement of medical practitioners [23,25], while others did not [24]. This variability makes it difficult to compare studies and be certain about what aspects of midwifery care are important. It is impossible to know, for instance, if it is the home-like setting that matters or the continuity of care. The variability between models of care is particularly important in this area of research because adverse outcomes are quite rare, which means study results often need to be pooled in order to reach statistical significance. This is impossible when the type of service being examined differs between studies.

Another difficulty in interpreting the data arises from the high transfer rates from birth centres to standard care seen in many studies. Some argue that this means existing criteria for admission to birth centre care are inadequate [21,24]. In Australia, however, national midwifery guidelines for referral and consultation have been developed [26] but to date, they have failed to allay concerns about the safety of midwifery-led care. Classifying risk and developing referral guidelines can only go so far towards assuring safety. Childbirth never comes with absolute assurances and the evidence base underlying service delivery (like many other health services) will never be rock solid. Because of the difficulties of producing irrefutable evidence on the safety of midwifery-led care, debates about it will continue.

### Is the lack of evidence enough to prevent reform?

In discussing how difficult decisions are made, health economist Anthony Culyer describes the 'weighing up' of different kinds of evidence including the more colloquial use of professionals' experiences when more than one profession is involved and stakeholders have conflicting interests [27]. In their recent analysis of the role of evidence in policy-making on obesity, Nathan et al. showed that it *is *possible to make progress in the absence of compelling evidence [28]. The authors found that in some cases when empirical evidence was lacking, policy-makers were prepared to make decisions based largely on opinion and ideas. However, importantly, they found that in more contentious policy areas which involve interventions that affect everyone and require the development of a national policy (such as food advertising to children), a lack of compelling evidence gave policy-makers good reason to avoid taking action. The lesson to be learnt from the Obesity Summit in NSW is that governments will need a substantial evidence base before they will take action in maternity services. Given the power of medical representative organisations in Australia, and their strong opposition to the extension of midwifery-led services on the grounds that it puts mothers and babies at risk, policy-makers are likely to take an extremely cautious approach.

### Where does this leave midwifery-led care?

Midwifery advocates' best hope for eventual system-wide changes to maternity care is to focus in the short term on local level reforms. This will allow them to include critics and sceptics in trials of midwifery care, thereby ensuring their concerns about safety are addressed and that new models are developed that are safe, effective and provide women with the choice they want.

There are several examples of successful programs where advocates have begun by trialling a new model of care, which has then gone on to become an established service. One such program is the St George Outreach Maternity Program (STOMP) in suburban Sydney. STOMP offers team midwifery for community-based antenatal clinic care, hospital intrapartum care and combined hospital and home-based postnatal care. This popular program caters for 720 women per year and can cover women who develop risk factors during their pregnancy through collaboration with obstetricians who also attend the clinic[29].

Other examples include the two successful small scale state-funded community midwifery programs currently operating in Australia: one in the Perth metropolitan area of Western Australia and the other in northern suburbs of Adelaide in South Australia. Both offer one-to-one care throughout all perinatal phases, with the Perth program providing mainly home birth services and the Adelaide program targeting young, Aboriginal or low socioeconomic status women. Midwifery care continues, with medical support, if women become high risk [29].

In 2005 in far north Queensland, the planned closure of a midwifery-led service at Mareeba Hospital was averted because of community protest. In response, the state government agreed to let the unit operate as a trial midwife-led birthing facility. Subsequent reviews commented on the high standard of care, quality and safety [30]. The Mareeba model has been commended because it addresses the major priorities for change outlined in the *Rebirthing *report [6]namely "poor outcomes for Aboriginal and Torres Strait Islander babies, care for women in rural and remote areas; and the dearth of post-birth care" [30] and serves as a clear example of the power of community-level advocates to overcome inadequacies in state-based services.

Another example of a local trial of midwifery-led care has been operating in rural Victoria. This service was designed to capture those women in the community not accessing antenatal care, especially very young women, but also those with drug or alcohol dependence or abuse, mental health problems, the homeless or those with previous experience of a neonatal death and/or difficult pregnancy and birthing [31]. While the Mareeba and rural Victorian trials may not yet have obtained a commitment to ongoing funding, such trials provide proof of the feasibility and value of these programs to the local community. Evidence of their successful operation may in turn be used as a powerful tool for influencing policy-makers' decisions to continue and/or expand these trials.

Although this incremental approach to system-wide reform may be frustratingly slow, it is much more likely to succeed. At the local level, clinicians from various professional backgrounds routinely work together. It is from this basis of collaboration and cooperation that alternative models of care that are safe and effective are most likely to emerge. Reforming services at the local level means system-wide reforms, such as facilitating independent midwifery practice, are less likely to be 'stonewalled' from the outset by critics using evidence, or at least doubts about it, to convince decision-makers that more caution is required. Advocates wishing to improve access to midwifery-led care need to resist the temptation to campaign for reform at a system-wide level and focus their attention instead on implementing change slowly, and at the local level.

## Summary

Advocates seeking to expand women's access to birth centres, or midwifery care in general, need to be mindful of the various obstacles to reform in maternity services. Although a lack of political will is often blamed when there is no progress on a system-wide level, it is important to recognise the underlying role that evidence plays in determining whether or not policy-makers are willing to implement changes. It is difficult to "prove" that midwifery care is safe because methodological holes in the evidence reported in the literature mean there will always be an element of doubt, to which critics will undoubtedly draw attention. Local examples of midwifery-led services that are feasible and popular are, we believe, the best form of evidence to persuade policy-makers of the need for change. Advocates should attempt to improve access to maternity-led care through a grass roots approach to reform. Then, region by region, the expansion of midwifery-led services in Australia may at last get underway.

## Competing interests

They author(s) declare they have no competing interests.

## Authors' contributions

AB and KF both contributed to the review of literature that forms the basis of this argument and the preparation of the manuscript. Both authors have read and approved the final manuscript.
